# A Rare Case of Hemophagocytic Lymphohistiocytosis in an Adult

**DOI:** 10.7759/cureus.23545

**Published:** 2022-03-27

**Authors:** Brian Behnke, Nikhilesh Srinivasan, Irene Soesilo, Ryan Spilman

**Affiliations:** 1 Internal Medicine, Sky Ridge Medical Center, Lone Tree, USA; 2 Internal Medicine, Presbyterian St. Luke's Medical Center, Denver, USA

**Keywords:** soluble cd25, mucositis, disseminated rash, cytotoxic t-cells, natural killer cells, persisting fever, ebv, genetic mutation, neutropenic fever, hemophagocytic lymphohistiocytosis

## Abstract

This case report involves an adult patient diagnosed with a rare disease, hemophagocytic lymphohistiocytosis (HLH). We will discuss the patient's clinical presentation, symptoms, and treatment. Due to the rarity of HLH being found in adults, we will break down the essential elements to recognize and diagnose this disease. We present this case to increase physician awareness of HLH occurring in adults. With timely recognition, more patients will be able to receive appropriate treatment, resulting in a decrease in mortality.

## Introduction

Hemophagocytic lymphohistiocytosis (HLH) is a life-threatening syndrome characterized by excessive immune activation. Uncontrolled, activated macrophages and T lymphocytes secrete high amounts of cytokines leading to multiorgan failure [1]. Though usually a disease diagnosed in children, adult cases of HLH are under-recognized and, therefore, undertreated [2]. The clinical presentation of HLH presents similarly to presentations of infection with severe sepsis, which can delay the diagnosis. HLH is aggressive and fatal in less than a few months if left untreated [2]. This case study is to help highlight the clinical presentation and diagnosis criteria of HLH in order to establish earlier recognition of the disease. By being able to recognize and consider HLH more efficiently, treatment can be initiated promptly, leading to less mortality.

## Case presentation

**Figure 1:**
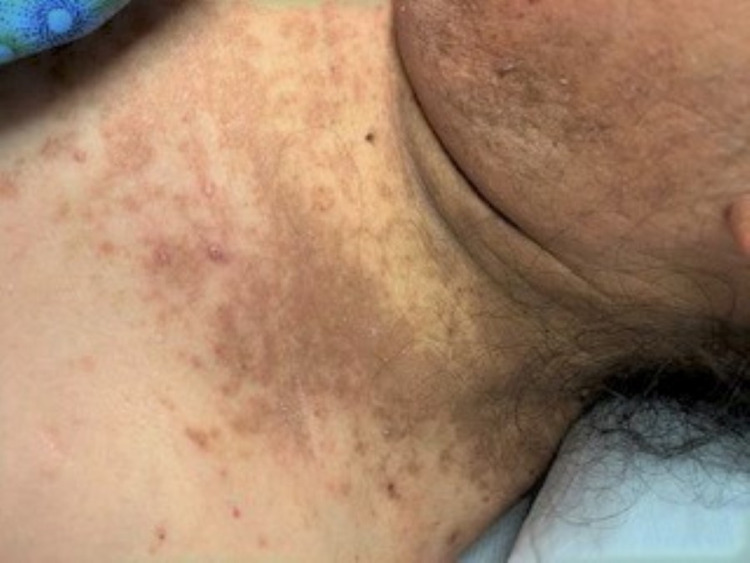
Photo of patient’s rash (left shoulder and neck)

**Figure 2:**
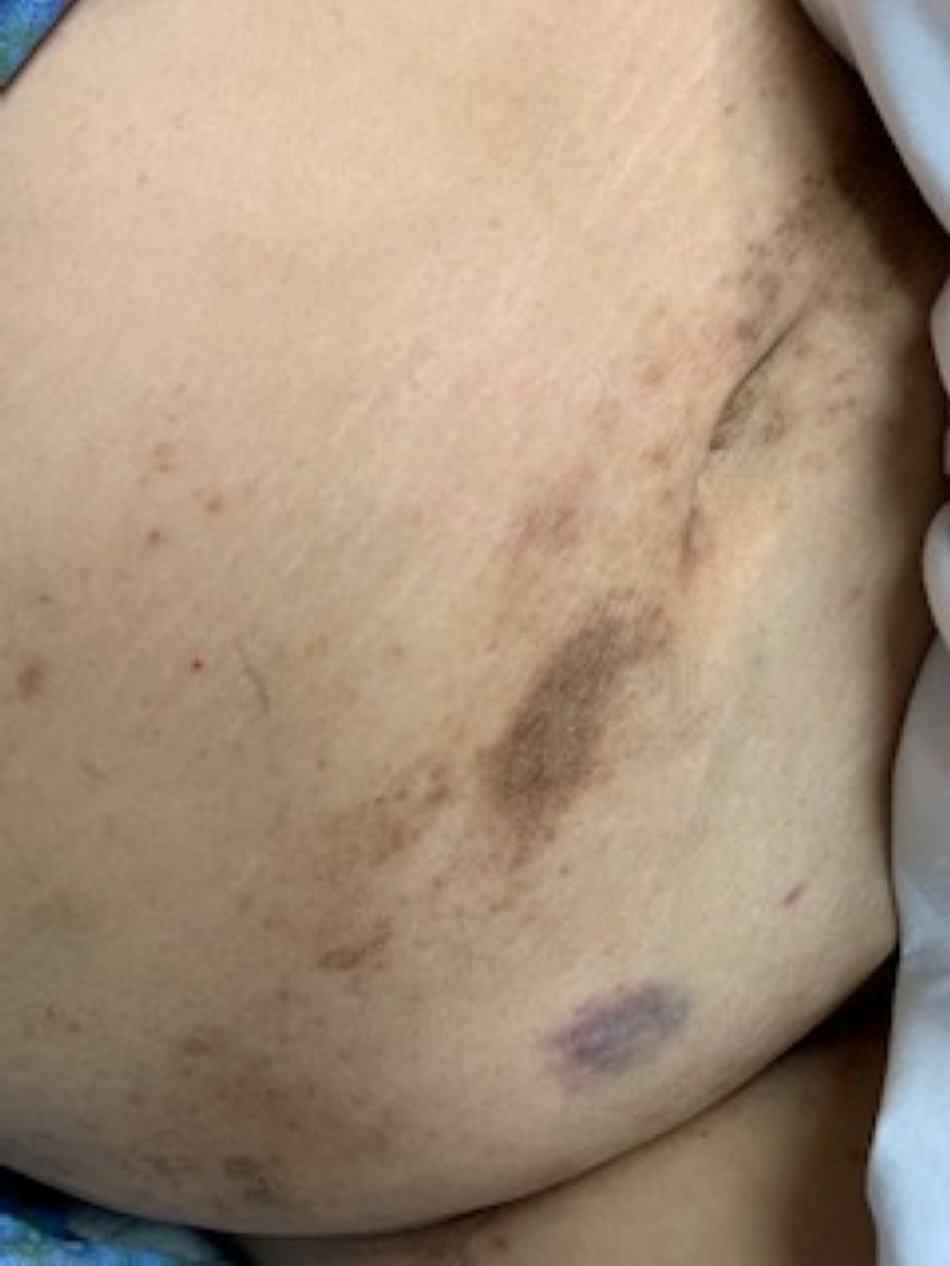
Photo of patient’s rash (abdomen)

A 59-year-old female with a medical history of rheumatoid arthritis and systemic lupus erythematosus (SLE) (on methotrexate and prednisone) was transferred from an out-of-state facility for neutropenic fever, mucositis, and a painful vesicular rash, with concerns of disseminated zoster (Figures 1-2). The patient’s mucositis was extremely painful and hemorrhagic, making it difficult for her to speak, eat, or drink. The rash extended from the patient’s forehead, neck, chest, abdomen, and posterior thighs.

The patient had a fever of 102.6^o^F and was tachycardia but normotensive. She had a WBC of 0.19× 10^9^/L with an absolute neutrophil count of 10× 10^9^/L, hemoglobin of 7.8 g/dL, and platelet count of 6 × 10^9^/L. Methotrexate levels were within normal limits. Bacterial and fungal blood cultures had no growth. A bone marrow biopsy was performed, which showed markedly hypocellular bone marrow. Viral polymerase chain reactions (PCRs) were drawn, which showed that Epstein-Barr virus (EBV) was positive at 2000 IU/mL. The patient was found to have hypertriglyceridemia of 294 mg/dL and elevated ferritin of >16500 ng/mL. Soluble CD25 was ordered, which returned with an extremely increased value of 27000 U/mL. These tests confirmed the diagnosis of hemophagocytic lymphohistiocytosis. The patient was started on etoposide, dexamethasone, and intrathecal chemotherapy with methotrexate according to the HLH-94 protocol.

The patient’s hospitalization was complicated due to her anemia with hemoglobin continually falling below 7.0g/dL and platelet count below 20 × 10^9^/L, requiring multiple transfusions of packed red blood cells and platelets. The patient’s mental status deteriorated causing her to be somnolent and difficult to arouse with occasional episodes of being fully alert and oriented. The patient routinely had intermittent fevers. A lumbar puncture was performed, which yielded no growth on cerebral spinal fluid culture as well as a negative panel. Computerized tomography of the head and magnetic resonance imaging of the brain showed no abnormalities. No growth on multiple sets of blood cultures was found. The patient also developed cavitary and noncavitary lesions in her right lung. A bronchoscopy was performed and cultures were positive for methicillin-resistant staphylococcus aureus (MRSA).

With treatment of her various medical complications and with etoposide, dexamethasone, and intrathecal chemotherapy, the patient's condition had become stable for her to return home. The HLH-94 protocol had helped her neutropenia improve. She was able to finish her intrathecal chemotherapy course in the hospital prior to discharge. She was arranged for close follow-up with a physician near her home who could continue to manage her antibiotics for her MRSA pneumonia and continue her etoposide and dexamethasone according to the HLH-94 protocol.

## Discussion

There are two classifications of HLH: familial and acquired. Familial, mainly seen in infants, involves a genetic mutation in the natural killer cell (NK) and cytotoxic T-cell function. Acquired HLH expresses the HLH phenotype without a genetic mutation. Activated lymphocytes are recruited to antigen-presenting cells, which causes a significant elevation in proinflammatory cytokine release into the circulation and systemically activated lymphocytes and macrophages. In acquired HLH, NK and cytotoxic T cells suffer permanent or transient dysfunction, which causes the inability to regulate the immune response. In both familial and acquired, the activated lymphocytes and macrophages target the immune system, resulting in hemophagocytosis and cytopenias [3-4].

HLH presents rapidly as symptoms seen within several days to weeks. Common findings include fever, hepatitis, rash, pulmonary dysfunction, cytopenia, coagulopathy, and neurological symptoms (seizures and decreased levels of consciousness, meningismus, or confusion) [5-6]. Although jaundice, hepatomegaly, and splenomegaly are common signs in children with the diagnosis, these symptoms are much less seen in adults [7]. The most common triggering factors of acquired HLH are infections and malignancies. Among infections, EBV is the most prevalent, incorporating 15.3% of 137 cases in a collaborative analysis across Germany [8].

**Figure 3:**
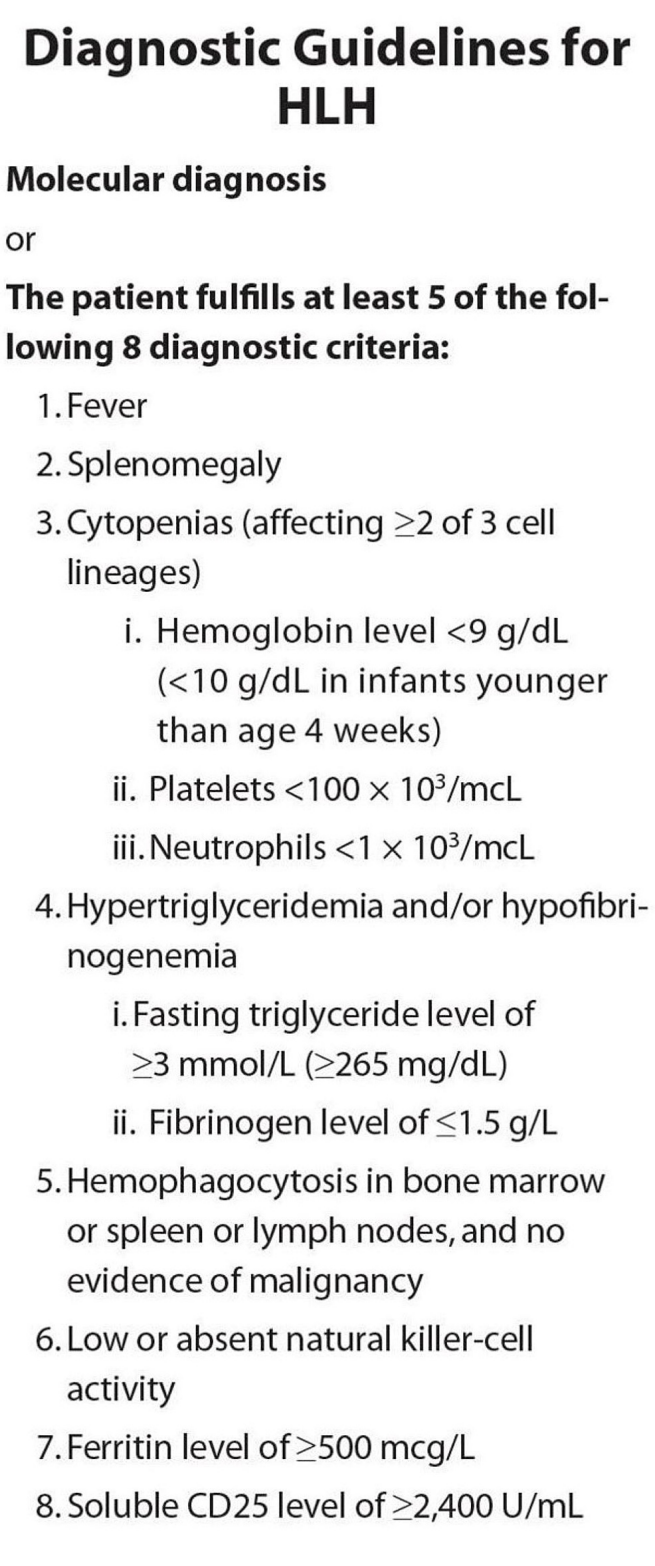
Guidelines for HLH diagnosis HLH: hemophagocytic lymphohistiocytosis

**Figure 4:**
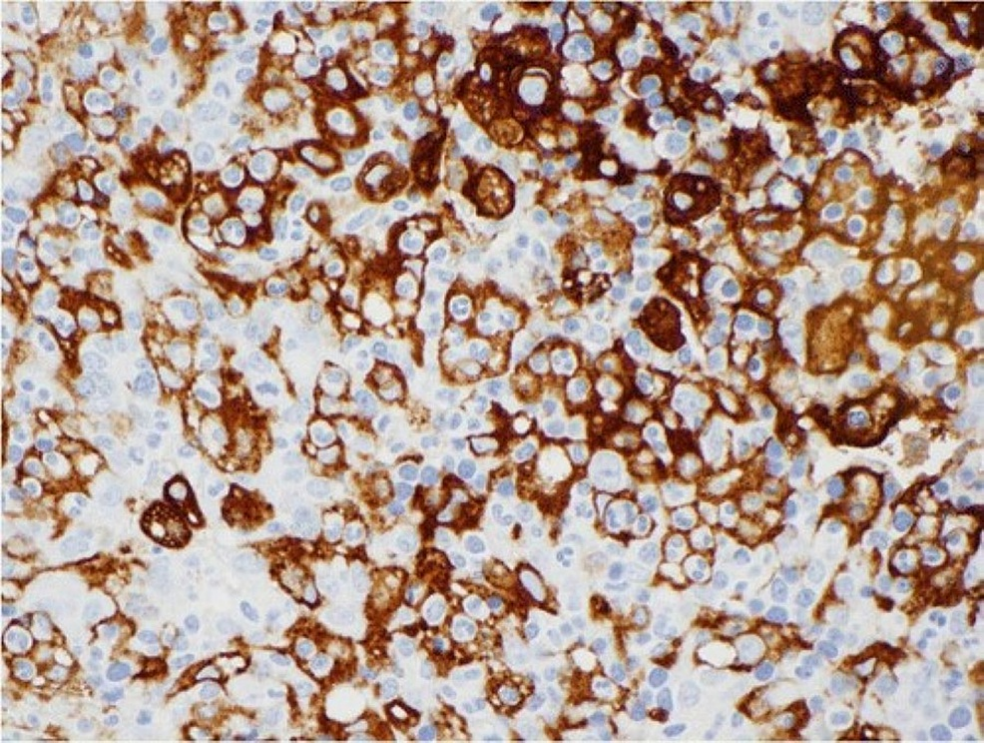
CD163 staining of the spleen of a patient diagnosed with HLH HLH: hemophagocytic lymphohistiocytosis

The diagnostic criteria of HLH involve a molecular diagnosis or meeting five of the eight criteria, including fever, splenomegaly, bicytopenia, hypertriglyceridemia and/or hypofibrinogenemia, hemophagocytosis in the bone marrow, low or absent NK cell activity, elevated ferritin levels, and elevated soluble CD25 levels (Figure 3) [5]. Hyperferritinemia and elevated levels of soluble CD25 are excellent predictors of cytokinemia as elevated ferritin levels are a reflector of activated macrophages and soluble CD25 is the gold standard of T-cell activation [9]. New modalities are being tested to help establish a diagnosis of HLH. Though not established as a diagnostic tool, CD163m, a receptor for hemoglobin-haptoglobin complexes and macrophage-activation marker, is showing promise as a new laboratory maker for HLH. CD163 staining of the spleen of a patient diagnosed with HLH is seen in Figure 4 [10].

The mortality rate of HLH was 95% within one to two months before HLH-directed therapy was initiated [10]. The most accepted treatment was been the HLH-94 protocol, however, this treatment method has been used in large prospective pediatric studies. There have been no prospective treatment trials in adults. The treatment regimen consists of an eight-week course of dexamethasone and etoposide. If neurological symptoms are present, intrathecal methotrexate is recommended. For relapsing disease, maintenance therapy is initiated with dexamethasone pulses, tacrolimus (less nephrotoxic), and etoposide until the patient is able to receive a stem cell transplant [4]. The HLH-94 protocol has increased patient survival to 54% with a median follow-up at 6.2 years [9].

## Conclusions

Hemophagocytic lymphohistiocytosis is a rare and deadly disease. Though most commonly seen in the pediatric population, HLH must be considered in adult patients with the common signs and symptoms being high, persistent fevers refractory to antibiotics. Treatment of this disease decreases mortality. If recognized early and treated using the HLH-94 protocol, HLH can become a much more manageable disease.
